# Association between HLA-B*13:01 and DIHS/DRESS due to dapson in a Japanese patients of pityriasis lichenoides et varioliformis acuta (PLEVA)

**DOI:** 10.1186/2045-7022-4-S3-P13

**Published:** 2014-07-18

**Authors:** Hideaki Watanabe, Erika Tonooka, Hirokazu Uno, Takeshi Ozeki, Taisei Mushiroda, Hirohiko Sueki

**Affiliations:** 1 Department of Dermatology, Showa University School of Medicine, Japan; 2 RIKEN Center for Integrative Medical Sciences, Research Group for Pharmacogenomics, Japan

## Background

Human leukocyte antigen (HLA)-B*13:01 and HLA-C*03:04, which have recently been reported to be strongly associated with dapson-induced DIHS/DRESS in patients suffering from leprosy in China.

## Observation

A 38-year old Japanese woman visited our dermatological department on June 24, 2013, with a three-day history of erythematous lesions on the trunk and extremities. She had been treated orally with 50 mg of diaminodiphenyl sulfone (dapson) daily for pityriasis lichenoides et varioliformis acuta (PLEVA) for 38 days. Physical examination on admission revealed fever (38.4℃), bilateral cervical lymphadenopathy, facial edema with erythema, and round erythematous and purpuric plaques on the trunk and extremities. Erythematous targetoid lesions were also found. Blood examination revealed an increased white blood cell count (13.4 ~ 109/l: normal 3.5-9 ~ 109/l), and mild liver dysfunction (alanine aminotransferase; 40 IU/l :normal 5-25 IU/l). A skin biopsy obtained from the patient's abdomen revealed acanthosis, hydropic and vacuolar degeneration of epidermal basal cells and upper dermal infiltration consisting of lymphocytes and neutrophils. No extensive epidermal necrosis was found. A lymphocyte transformation test (LTT) for dapson was positive (stimulation index (SI) = 5.3, cut-off for LTT, SI = 1.8). These findings fulfilled the criteria for drug-induced hypersensitivity syndrome (DIHS)/drug reaction with eosinophilia and systemic symptoms (DRESS), and dapson therapy was discontinued. The patient was started on a course of systemic prednisolone (1.0 mg/kg daily). The dose was tapered over the next 70 days in line with the improvement of clinical symptoms. Both human herpesvirus-6 and cytomegalovirus reactivation had not been seen during the course. Human leukocyte antigen (HLA)-B*13:01 and HLA-C*03:04 were identified in this patient.

## Conclusion

Our examination indicated that these HLA alleles are not only related to DIHS/DRESS in patients with leprosy treated with dapson, but also to DIHS/DRESS in patients treated with dapson for other inflammatory diseases including PLEVA.

